# Dietary total antioxidant capacity from different assays in relation to serum C-reactive protein among young Japanese women

**DOI:** 10.1186/1475-2891-11-91

**Published:** 2012-10-30

**Authors:** Satomi Kobayashi, Kentaro Murakami, Satoshi Sasaki, Kazuhiro Uenishi, Mitsuyo Yamasaki, Hitomi Hayabuchi, Toshinao Goda, Jun Oka, Keiko Baba, Kazuko Ohki, Reiko Watanabe, Yoshiko Sugiyamama

**Affiliations:** 1 Department of Social and Preventive Epidemiology, Graduate School of Medicine, the University of Tokyo, Tokyo, Japan; 2 Department of Social and Preventive Epidemiology, School of Public Health, the University of Tokyo, 7-3-1 Hongo, Bunkyo-ku, Tokyo 113-0033, Japan; 3 Laboratory of Physiological Nutrition, Kagawa Nutrition University, Saitama, Japan; 4 Department of Health and Nutrition Science, Faculty of Health and Social Welfare Science, Nishikyushu University, Saga, Japan; 5 Department of Human Environmental Science, Fukuoka Women’s University, Fukuoka, Japan; 6 Department of Nutrition, School of Food and Nutritional Sciences, University of Shizuoka, Shizuoka, Japan; 7 Department of Nutrition, Faculty of Home Economics, Tokyo Kasei University, Tokyo, Japan; 8 Department of Kids Culture Design Center, Mie Chukyo University, Mie, Japan; 9 Graduate School of Science for Living System, Showa Women’s University, Tokyo, Japan; 10 Department of Health and Nutrition, University of Niigata Prefecture, Niigata, Japan; 11 Department of Nutrition Management, Faculty of Health and Nutrition, Minami Kyushu University, Miyazaki, Japan

**Keywords:** Dietary total antioxidant capacity, Serum C-reactive protein, Diet history questionnaire, Young Japanese women, Epidemiology

## Abstract

**Background:**

The association between dietary total antioxidant capacity (TAC) from different assays and serum C-reactive protein (CRP) has not been assessed in non-Western populations. We examined the association between dietary TAC and serum CRP concentration in young Japanese women using different four TAC assays.

**Methods:**

The subjects were 443 young Japanese women aged 18–22 years. Dietary TAC was assessed with a self-administered diet history questionnaire and the TAC value of each food using the following four assays: ferric reducing ability of plasma (FRAP); oxygen radical absorbance capacity (ORAC); Trolox equivalent antioxidant capacity (TEAC); and total radical-trapping antioxidant parameter (TRAP). Serum CRP concentrations were measured by highly sensitive nephelometry.

**Results:**

The major contributor to dietary TAC was green, barley, and oolong tea (FRAP: 53%, ORAC: 45%, TEAC: 36%, and TRAP: 44%). The prevalence of elevated CRP concentrations (≥ 1 mg/L) was 5.6%. TAC from FRAP was inversely associated with serum CRP concentrations (adjusted odds ratio [OR] for elevated CRP concentration in high [compared with low] dietary TAC group: 0.39 [95% confidence interval (CI): 0.16-0.98]; *P* = 0.04). TAC from ORAC was inversely associated with CRP, although the association was not significant (OR: 0.48 [95% CI: 0.20-1.14]; *P* = 0.10). TAC from TEAC was inversely associated with CRP (OR: 0.32 [95% CI: 0.12-0.82]; *P* = 0.02), as was TAC from TRAP (OR: 0.31 [95% CI: 0.12-0.81]; *P* = 0.02).

**Conclusions:**

Dietary TAC was inversely associated with serum CRP concentration in young Japanese women regardless of assay. Further studies are needed in other populations to confirm these results.

## Background

C-reactive protein (CRP) is produced by hepatocytes as part of the acute-phase response and represents a sensitive and non-specific marker of inflammation [1]. CRP is associated with cardiovascular disease, type 2 diabetes mellitus, and cancer [2], and identification of the factors associated with serum CRP concentration is important to their prevention. Dietary factors represent one major modifiable factor related to CRP, and several previous studies have shown that increasing the intake of antioxidant nutrients (e.g., vitamin C and vitamin E) or foods (e.g., tea, fruits, and vegetables) is associated with decreased CRP concentrations [2-4]. In other studies, however, these single nutrients or foods were shown to have no effect [2,5-7]. Accumulating mechanistic and epidemiological data suggest that antioxidants act not only individually but also co-operatively, and in some cases synergistically [8]. The effect of complex combinations of antioxidant nutrients and foods on CRP concentrations therefore warrants investigation.

Recently, the concept of dietary total antioxidant capacity (TAC) was proposed to assess the combined effect of multiple antioxidants [9]. Previous observational studies [10-12] and an interventional study [13] showed that dietary TAC was inversely related to CRP among Italian, Spanish, and Greek populations. Given that dietary habits vary by country - vegetables commonly consumed in Japan differ from those consumed in Italy, for example [14,15] - the food source of dietary TAC at the intake level may also differ, and the association should therefore be evaluated in other countries, including Japan. Further, because CRP concentration tends to increase with age [16], maintaining concentrations as low as possible in younger age is an important preventive measure. Evaluation of the association between dietary TAC and CRP among young adult populations is therefore important.

Here, we estimated dietary TAC from different assays in young Japanese women and examined the association between dietary TAC and serum CRP concentration using these assays.

## Methods

### Study population

Details of the study population have been reported elsewhere [5]. Briefly, the present study was based on a multi-center survey conducted from February to March 2006 among female dietetic students from 10 institutions in Japan. The study protocol was approved by the Ethics Committee of the National Institutes of Health and Nutrition, and written informed consent was obtained from each subject, and also from a parent for subjects aged less than 20 years. A total of 474 women took part. We excluded subjects whose CRP concentrations had not been measured (n = 22), those with CRP concentrations ≥10 mg/L (n = 2) on the basis that such high concentrations were likely caused by infection or an underlying medical problem not related to diet [17], and those aged <18 or ≥23 years (n = 7). The final sample thus comprised 443 women aged 18–22 years. All women were free from diabetes, hypertension, and cardiovascular disease, and none reported extremely low (less than half the energy requirement for the lowest physical activity category (< 775 (1550 × 0.5) kcal/day)) or high (more than 1.5 times the energy requirement of the highest physical activity category (> 3450 (2300 × 1.5) kcal/day)) energy intake with reference to the Recommended Dietary Allowance for Japanese [5,18,19].

### Dietary assessment

Dietary habits during the preceding month were assessed using a previously validated, self-administered comprehensive diet history questionnaire (DHQ) [20,21]. Responses to the DHQ as well as to an accompanying lifestyle questionnaire were checked at least twice for completeness, and reviewed with the subject when necessary to ensure the clarity of answers. Estimates of dietary intake for a total of 150 food items, energy, and n-3 polyunsaturated fatty acid (PUFA) were calculated using an ad hoc computer algorithm for the DHQ based on the Standard Tables of Food Composition in Japan [22]. Although dietary supplement use was queried in the DHQ, intake from supplements was not included in analyses due to the lack of a reliable composition table of dietary supplements in Japan.

### Estimation of dietary TAC

To calculate dietary TAC, a TAC value was assigned to each food item in the DHQ. Among the 150 food items in the DHQ, 54 foods were determined to contain no TAC value because they contained no or only trace amount of antioxidant nutrients such as carotenes, vitamin C, or vitamin E (e.g., animal foods and refined foods). We therefore searched for the analytical TAC value of the remaining 96 foods in the DHQ using the following four assays: ferric reducing ability of plasma (FRAP); oxygen radical absorbance capacity (ORAC); Trolox equivalent antioxidant capacity (TEAC); and total radical-trapping antioxidant parameter (TRAP).

Studies reporting the analytical TAC value of foods were identified by searching the PubMed database, which resulted in many TAC values for assignment to specific foods (about 70% of 96 foods). If we could not obtain a TAC value from PubMed, we searched the database of one journal, *Food Chemistry*, which was frequently referred to in the papers obtained from the PubMed search. The TAC values of four foods for ORAC and 11 foods for TEAC were obtained from this journal database only [23-28]. We therefore used papers in the literature reporting TAC values of food in analytical data for FRAP [15,29-34], ORAC [14,23,24,35-45], TEAC [15,25-28,33,46-49], and TRAP [15,33]. TAC values were expressed as mmol Fe^2+^/100 g of food for FRAP or as mmol of Trolox equivalent (mmol TE)/100 g of food for ORAC, TEAC, and TRAP.

When the analytical TAC value for a specific food could be obtained, the mean TAC value was calculated by weighting the number of foods analyzed in each study, and the mean value was assigned (analytical value; FRAP: n = 40, ORAC: n = 23, TEAC: n = 22, and TRAP: n = 21). When the analytical TAC value for a specific food could not be obtained, the mean value of similar foods or a different form of the same food was assigned (substituted value; FRAP: n = 22, ORAC: n = 20, TEAC: n = 22, and TRAP: n = 20). For fresh foods, if the TAC value of the food was obtained in dry matter only, the TAC value was calculated using the dry matter value and the proportion of water in the fresh food. For processed foods, if we obtained the TAC values of the ingredient foods, TAC values were calculated by the values of the ingredient foods and their proportion of the total food component. When we obtained a TAC value by these calculations, this value was assigned (calculated value; FRAP: n = 18, ORAC: n = 27, TEAC: n = 29, and TRAP: n = 17). The remaining foods were not assigned a TAC value because no value could be assigned using any of the above steps (FRAP: n = 16, ORAC: n = 26, TEAC: n = 23, and TRAP: n = 38). Of the 96 foods for which TAC values were sought, 12 could not be assigned a value by any of the four methods. Values for the remaining 84 food items used to calculate dietary TAC in this study are presented in Table 1. Dietary TAC was estimated based on intake and the TAC value of each food.

**Table 1:** TAC values of food items included in the diet history questionnaire and the contribution (%) of dietary TAC among 443 Japanese women aged 18–22 years

**Food group and item†‡**	**FRAP**			**ORAC**			**TEAC**			**TRAP**		
	**TAC(mmol Fe**^**2+**^**/100 g)**		Contribution*(%)	**TAC(mmol TE/100 g)**		Contribution*(%)	**TAC(mmol TE/100 g)**		Contribution*(%)	**TAC(mmol TE/100 g)**		Contribution*(%)
Non-alcoholic beverages	-		77.77 (66.79-85.02)	-		67.18 (55.23-77.39)	-		63.66 (51.56-74.41)	-		77.65 (66.16-85.78)
Green, barley, and oolong tea (including other Chinese tea)	1.41	||	57.31 (36.17-73.16)	1.86	¶	46.97 (28.17-63.41)	0.54	||	36.75 (19.33-52.92)	0.69	||	46.47 (23.16-65.41)
Coffee	3.23	§	3.16 (0.00-17.16)	4.24	§	2.68 (0.00-14.91)	3.14	§	6.22 (0.00-27.48)	5.60	§	10.15 (0.00-41.59)
Black tea	0.97	§	0.00 (0.00-5.20)	1.48	¶	0.00 (0.00-4.74)	0.36	§	0.00 (0.00-3.08)	0.49	§	0.00 (0.00-4.07)
Cocoa	0.80	§	0.00 (0.00-1.70)	3.03	¶	0.00 (0.00-4.07)	1.41	¶	0.00 (0.00-5.40)	0.45	¶	0.00 (0.00-1.68)
Fruit juice (100%)	0.62	||	0.00 (0.00-0.94)	0.64	||	0.00 (0.00-0.62)	0.27	||	0.00 (0.00-0.63)	0.23	||	0.00 (0.00-0.45)
Vegetable juice	0.38	||	0.00 (0.00-0.85)	0.48	§	0.00 (0.00-0.62)	0.17	||	0.00 (0.00-0.61)	0.16	||	0.00 (0.00-0.55)
Tomato juice	0.48	§	0.00 (0.00-0.00)	0.43	§	0.00 (0.00-0.00)	0.17	||	0.00 (0.00-0.00)	0.13	||	0.00 (0.00-0.00)
Fruit juice, excluding 100% juice	0.31	¶	0.00 (0.00-0.00)	0.32	¶	0.00 (0.00-0.00)	0.14	¶	0.00 (0.00-0.00)	0.11	¶	0.00 (0.00-0.00)
Vegetables	-		7.50 (4.56-12.13)	-		11.13 (7.29-16.19)	-		8.61 (5.41-12.65)	-		8.29 (5.11-13.38)
Green leafy vegetables	1.08	||	2.27 (1.03-4.23)	1.53	||	2.02 (0.95-3.72)	0.69	||	2.28 (1.18-4.56)	0.48	||	1.50 (0.75-3.15)
Cabbage	0.45	§	0.69 (0.30-1.19)	0.52	||	0.50 (0.22-0.85)	0.12	||	0.30 (0.13-0.54)	0.28	||	0.66 (0.29-1.25)
Chinese cabbage	0.45	||	0.46 (0.22-1.09)	0.52	||	0.33 (0.17-0.76)	0.12	||	0.21 (0.11-0.46)	0.28	||	0.44 (0.22-1.14)
Broccoli	0.96	§	0.45 (0.20-1.18)	1.48	||	0.45 (0.21-1.13)	0.30	||	0.24 (0.11-0.61)	0.31	||	0.25 (0.09-0.61)
Onions	0.29	§	0.37 (0.19-0.70)	1.22	||	0.99 (0.52-1.78)	0.18	||	0.39 (0.19-0.71)	0.24	||	0.49 (0.24-0.94)
Radishes	0.31	||	0.30 (0.15-0.61)	1.50	||	0.91 (0.47-1.81)	0.55	||	0.86 (0.47-1.73)	0.66	||	0.99 (0.50-2.05)
Green peppers	1.02	§	0.24 (0.10-0.48)	0.93	§	0.14 (0.06-0.27)	0.84	||	0.34 (0.13-0.64)	0.55	||	0.21 (0.07-0.40)
Mushrooms	0.22	||	0.19 (0.09-0.35)	0.65	||	0.35 (0.16-0.63)	0.49	||	0.70 (0.33-1.29)	0.63	||	0.88 (0.39-1.55)
Tomatoes	0.27	§	0.18 (0.08-0.40)	0.39	§	0.16 (0.07-0.37)	0.17	§	0.18 (0.08-0.39)	0.13	§	0.14 (0.05-0.31)
Lotus root	1.58	||	0.17 (0.00-0.36)	2.10	||	0.14 (0.00-0.31)	-		-	-		-
Lettuce	0.27	§	0.11 (0.04-0.26)	0.32	§	0.08 (0.03-0.20)	0.13	§	0.08 (0.04-0.20)	0.23	§	0.14 (0.05-0.37)
Carrots	0.10	||	0.10 (0.05-0.20)	0.33	||	0.21 (0.11-0.40)	0.04	||	0.07 (0.03-0.12)	0.07	||	0.12 (0.05-0.20)
Other salted pickles	0.36	¶	0.09 (0.03-0.27)	1.29	¶	0.21 (0.07-0.58)	0.31	¶	0.13 (0.05-0.35)	0.45	¶	0.18 (0.06-0.52)
Pumpkins	0.11	||	0.04 (0.02-0.08)	0.48	||	0.12 (0.04-0.23)	0.37	||	0.24 (0.09-0.46)	0.00	||	0.00 (0.00-0.00)
Eggplants	0.18	||	0.03 (0.00-0.09)	0.25	||	0.02 (0.00-0.07)	0.11	||	0.03 (0.00-0.09)	0.28	||	0.06 (0.00-0.21)
Cucumbers	0.05	§	0.01 (0.01-0.04)	0.21	§	0.04 (0.01-0.09)	0.04	§	0.02 (0.01-0.05)	0.00	§	0.00 (0.00-0.00)
Salted pickled plums	0.45	||	0.01 (0.00-0.04)	3.62	¶	0.07 (0.00-0.22)	0.47	¶	0.03 (0.00-0.08)	0.75	¶	0.04 (0.00-0.12)
Cauliflower	0.80	§	0.00 (0.00-0.00)	0.85	||	0.00 (0.00-0.00)	0.11	||	0.00 (0.00-0.00)	0.16	||	0.00 (0.00-0.00)
Burdock	-		-	5.22	||	0.87 (0.39-1.45)	-		-	-		-
Bean sprouts	-		-	1.72	||	0.38 (0.19-0.88)	0.15	||	0.09 (0.04-0.20)	-		-
Wakame and hijiki seaweed	-		-	0.45	¶	0.21 (0.10-0.40)	0.15	¶	0.18 (0.09-0.35)	-		-
Laver (dried, edible seaweed)	-		-	6.11	||	0.03 (0.02-0.08)	2.30	||	0.03 (0.02-0.08)	-		-
Fruits	-		3.67 (1.75-6.55)	-		5.92 (3.05-10.17)	-		3.12 (1.42-5.89)	-		2.49 (1.10-4.40)
Oranges	0.87	||	1.01 (0.36-2.62)	1.74	||	1.26 (0.47-3.15)	0.31	||	0.59 (0.22-1.46)	0.36	||	0.65 (0.23-1.67)
Strawberries	2.16	§	0.97 (0.26-2.10)	3.67	§	1.03 (0.30-2.20)	1.69	§	1.26 (0.32-2.59)	0.86	§	0.60 (0.12-1.35)
Apples	0.39	§	0.25 (0.06-0.69)	2.80	§	1.18 (0.30-2.89)	0.15	§	0.17 (0.04-0.40)	0.19	§	0.19 (0.04-0.51)
Bananas	0.19	§	0.08 (0.00-0.21)	0.74	§	0.21 (0.00-0.52)	0.06	§	0.04 (0.00-0.11)	0.11	§	0.07 (0.00-0.20)
Kiwi fruits	0.95	§	0.00 (0.00-0.30)	0.83	§	0.00 (0.00-0.17)	0.23	§	0.00 (0.00-0.12)	0.23	§	0.00 (0.00-0.10)
Canned fruits	0.42	¶	0.00 (0.00-0.08)	1.32	¶	0.00 (0.00-0.17)	0.19	¶	0.00 (0.00-0.07)	0.22	¶	0.00 (0.00-0.07)
Raisins	1.28	§	0.00 (0.00-0.07)	5.75	§	0.00 (0.00-0.20)	0.66	§	0.00 (0.00-0.06)	0.62	§	0.00 (0.00-0.04)
Grapes	0.78	§	0.00 (0.00-0.00)	1.29	§	0.00 (0.00-0.00)	0.32	§	0.00 (0.00-0.00)	0.20	§	0.00 (0.00-0.00)
Persimmons	0.49	§	0.00 (0.00-0.00)	0.74	§	0.00 (0.00-0.00)	6.86	¶	0.00 (0.00-0.00)	-		-
Peaches	0.24	§	0.00 (0.00-0.00)	1.92	§	0.00 (0.00-0.00)	0.17	§	0.00 (0.00-0.00)	0.15	§	0.00 (0.00-0.00)
Pears	0.22	||	0.00 (0.00-0.00)	1.80	||	0.00 (0.00-0.00)	0.22	||	0.00 (0.00-0.00)	0.39	||	0.00 (0.00-0.00)
Melons	0.18	§	0.00 (0.00-0.00)	0.24	§	0.00 (0.00-0.00)	0.09	§	0.00 (0.00-0.00)	0.10	§	0.00 (0.00-0.00)
Watermelons	0.05	§	0.00 (0.00-0.00)	0.17	§	0.00 (0.00-0.00)	0.07	§	0.00 (0.00-0.00)	0.05	§	0.00 (0.00-0.00)
Sugar and confectioneries	-		2.58 (1.38-5.58)	-		2.94 (1.56-6.00)	-		3.73 (2.02-7.63)	-		1.11 (0.56-2.55)
Chocolate	3.96	§	1.94 (0.98-4.63)	7.16	§	2.26 (1.13-5.28)	3.62	§	3.06 (1.54-6.83)	1.16	§	0.93 (0.42-2.33)
Cookies and biscuits	0.42	||	0.11 (0.05-0.24)	-		-	-		-	-		-
Japanese sweets with azuki beans	0.54	¶	0.09 (0.03-0.16)	0.42	¶	0.04 (0.01-0.08)	0.29	¶	0.08 (0.02-0.14)	0.18	¶	0.05 (0.01-0.09)
Jam and marmalade	0.71	¶	0.04 (0.00-0.13)	1.57	¶	0.05 (0.00-0.18)	0.40	¶	0.03 (0.00-0.13)	0.25	¶	0.02 (0.00-0.08)
Potato chips	0.59	§	0.04 (0.00-0.09)	2.09	¶	0.09 (0.00-0.21)	0.25	¶	0.03 (0.00-0.07)	0.26	¶	0.02 (0.00-0.07)
Japanese bread with a sweet filling	0.03	¶	0.03 (0.01-0.07)	0.25	¶	0.18 (0.07-0.36)	0.13	¶	0.24 (0.10-0.47)	-		-
Doughnuts	0.15	||	0.00 (0.00-0.09)	-		-	-		-	-		-
Pancakes	0.15	||	0.00 (0.00-0.02)	-		-	-		-	-		-
Jellies	0.01	||	0.00 (0.00-0.01)	-		-	-		-	-		-
Cereals	-		1.63 (0.96-3.33)	-		1.21 (0.41-3.00)	-		4.88 (3.25-7.86)	-		7.34 (4.22-12.05)
Well-milled rice	0.02	§	0.38 (0.20-0.68)	-			0.10	¶	3.21 (1.60-5.50)	0.18	¶	5.36 (2.39-10.26)
White bread	0.20	§	0.31 (0.16-0.67)	-		-	-		-	-		-
Japanese noodles (buckwheat and Japanese wheat noodles)	0.12	¶	0.18 (0.04-0.38)	0.65	¶	0.62 (0.14-1.31)	0.19	¶	0.49 (0.11-1.00)	0.12	¶	0.28 (0.05-0.63)
Spaghetti	0.03	§	0.05 (0.02-0.11)	-		-	0.08	¶	0.22 (0.07-0.46)	0.06	¶	0.15 (0.05-0.36)
Pizza	0.20	§	0.00 (0.00-0.15)	-		-	-		-	-		-
Cornflakes	0.93	§	0.00 (0.00-0.00)	2.36	§	0.00 (0.00-0.00)	0.22	§	0.00 (0.00-0.00)	0.18	§	0.00 (0.00-0.00)
Brown rice	0.27	§	0.00 (0.00-0.00)	1.14	¶	0.00 (0.00-0.00)	0.18	¶	0.00 (0.00-0.00)	0.22	¶	0.00 (0.00-0.00)
Half-milled rice	0.21	¶	0.00 (0.00-0.00)	0.60	¶	0.00 (0.00-0.00)	0.14	¶	0.00 (0.00-0.00)	0.20	¶	0.00 (0.00-0.00)
Well-milled rice mixed with barley	0.15	¶	0.00 (0.00-0.00)	0.39	¶	0.00 (0.00-0.00)	0.10	¶	0.00 (0.00-0.00)	0.14	¶	0.00 (0.00-0.00)
70% milled rice	0.13	¶	0.00 (0.00-0.00)	0.25	¶	0.00 (0.00-0.00)	0.12	¶	0.00 (0.00-0.00)	0.18	¶	0.00 (0.00-0.00)
Well-milled rice with germ	0.10	¶	0.00 (0.00-0.00)	0.13	¶	0.00 (0.00-0.00)	0.11	¶	0.00 (0.00-0.00)	0.18	¶	0.00 (0.00-0.00)
Oils	-		0.98 (0.56-1.62)	-		-	-		0.36 (0.24-0.53)	-		-
Oil used during cooking	0.44	¶	0.43 (0.28-0.65)	-		-	0.22	||	0.36 (0.24-0.53)	-		-
Mayonnaise	1.08	§	0.26 (0.00-0.62)	-		-	-		-	-		-
Margarine	1.05	§	0.05 (0.00-0.16)	-		-	-		-	-		-
Salad dressing	0.25	§	0.05 (0.00-0.16)	-		-	-		-	-		-
Nuts and pulses	-		0.93 (0.53-1.65)	-		3.49 (2.11-5.97)	-		7.70 (4.49-12.55)	-		0.07 (0.00-0.21)
Natto (fermented soybeans)	0.40	¶	0.22 (0.07-0.69)	2.27	¶	0.82 (0.28-2.50)	2.05	¶	2.02 (0.61-5.81)	-		-
Tofu (soybean curd)	0.09	||	0.12 (0.05-0.25)	0.79	¶	0.66 (0.30-1.42)	0.71	¶	1.55 (0.69-3.37)	-		-
Miso for miso soup	0.31	¶	0.12 (0.04-0.23)	1.72	¶	0.43 (0.16-0.77)	1.55	¶	1.03 (0.33-1.94)	-		-
Boiled beans	0.34	¶	0.06 (0.00-0.13)	2.09	¶	0.23 (0.00-0.49)	0.96	¶	0.28 (0.00-0.62)	-		-
Tofu (soybean curd) products	0.36	¶	0.00 (0.00-0.13)	2.05	¶	0.00 (0.00-0.47)	1.85	¶	0.00 (0.00-1.13)	-		-
Peanuts	1.20	§	0.00 (0.00-0.10)	3.17	§	0.00 (0.00-0.17)	0.48	§	0.00 (0.00-0.07)	0.33	§	0.00 (0.00-0.05)
Other nuts	0.84	||	0.00 (0.00-0.08)	4.69	||	0.00 (0.00-0.27)	4.76	¶	0.00 (0.00-0.71)	1.08	||	0.00 (0.00-0.16)
Miso as seasoning	0.31	¶	0.00 (0.00-0.00)	1.72	¶	0.00 (0.00-0.00)	1.55	¶	0.00 (0.00-0.00)	-		-
Seasonings	-		0.79 (0.42-1.30)	-		0.33 (0.19-0.53)	-		0.74 (0.42-1.14)	-		0.00 (0.00-0.04)
Soy sauce	1.68	||	0.75 (0.39-1.25)	1.06	¶	0.31 (0.17-0.48)	0.96	¶	0.71 (0.40-1.12)	-		-
Tomato ketchup	0.37	§	0.00 (0.00-0.06)	0.58	§	0.00 (0.00-0.05)	0.15	||	0.00 (0.00-0.04)	0.17	||	0.00 (0.00-0.04)
Potatoes	-		0.40 (0.22-0.73)	-		1.30 (0.73-2.28)	-		0.36 (0.19-0.63)	-		0.24 (0.11-0.48)
Sweet potatoes, yams, and taro	0.21	||	0.13 (0.07-0.27)	1.08	||	0.43 (0.22-0.86)	0.09	§	0.09 (0.05-0.18)	-		-
Potatoes	0.13	§	0.13 (0.06-0.28)	0.67	§	0.42 (0.21-0.89)	0.08	§	0.13 (0.07-0.28)	0.09	§	0.00 (0.06-0.33)
French fries	0.26	§	0.06 (0.00-0.13)	1.24	¶	0.17 (0.00-0.39)	0.15	¶	0.05 (0.00-0.12)	0.16	¶	0.05 (0.00-0.13)
Konnyaku (devil’s tongue jelly)	0.04	¶	0.01 (0.00-0.01)	0.20	¶	0.03 (0.01-0.04)	0.01	¶	0.00 (0.00-0.01)	-		-
Alcoholic beverages	-		0.00 (0.00-0.00)	-		0.00 (0.00-0.00)	-		0.00 (0.00-0.00)	-		0.00 (0.00-0.00)
Wine	1.41	§	0.00 (0.00-0.00)	1.17	§	0.00 (0.00-0.00)	0.63	§	0.00 (0.00-0.00)	0.82	§	0.00 (0.00-0.00)
Whiskey	0.25	§	0.00 (0.00-0.00)	-		-	0.15	§	0.00 (0.00-0.00)	0.19	§	0.00 (0.00-0.00)
Beer	0.24	§	0.00 (0.00-0.00)	-		-	0.10	§	0.00 (0.00-0.00)	0.00	§	0.00 (0.00-0.00)

*TAC* total antioxidant capacity, *FRAP* ferric reducing ability of plasma, *ORAC* oxygen radical absorbance capacity, *TEAC* Trolox equivalent antioxidant capacity, *TRAP* total radical-trapping antioxidant parameter, *TE* Trolox equivalent.

* Values are medians (interquartile ranges).

† These 84 food items from the 150 items in the diet history questionnaire were used in the present study. Among the remaining 66 food items, the following 54 were determined to contain no TAC value: all 15 items in fish and shellfish (dried fish; small fish with bones; canned tuna; eel; white meat fish; oily fish; red meat fish; ground fish meat products; shrimp and crab; squid and octopus; oysters: other shellfish; fish eggs; boiled fish and shellfish in soy sauce; and salted fish intestines); all 7 items in meat (ground beef and pork; chicken; pork; beef; liver; ham and sausages; and bacon); all 9 items in dairy products (full-fat milk; low-fat milk; skim milk; sweetened yogurt; nonsweetened yogurt; moderately sweetened yogurt; cheese; cottage cheese; and cream or creamer added to coffee); 5 items in other animal foods (ice cream [regular]; ice cream [premium]; ice cream [unspecified varieties]; butter; and eggs); and 18 items in other foods (sugar for coffee and black tea; sugar used during cooking; artificial sweeteners; candies, caramels, and chewing gum; non-oil dressings; table salt; salt used during cooking; corn soup; Chinese soup; soup consumed with noodles; water for miso soup; sake; shochu; shochu mixed with water or a carbonated beverage; lactic acid bacteria beverages; cola and sugar-sweetened soft drinks [including sports drinks]; sugar-free soft drinks and diet cola; and drinking water. The following 12 items could not assigned a TAC value for all four methods: instant noodles; Chinese noodles; butter roll; croissant; Japanese-style pancakes; rice crackers; snacks made from wheat flour; Japanese sweets without azuki beans; cakes; curry or stew roux and meat sauce; nutritional supplement drinks; and nutritional supplement bars.

‡ Food items were categorized into food groups according to our previous study [20]. Tomato ketchup and soy sauce were included in seasonings. Food groups and food items were listed in descending order according to the contribution to FRAP. Four food items not having a FRAP value were listed in descending order according to their contribution to ORAC.

§ TAC value was assigned an analytical value.

|| TAC value was assigned a substituted value.

¶ TAC value was assigned a calculated value.

### Serum CRP concentrations

Peripheral blood samples were obtained from subjects after an overnight fast. Blood was collected in evacuated tubes containing no additives, allowed to clot, and centrifuged at 3000 × *g* for 10 min at room temperature to separate serum. Blood samples were transported at −20°C to a laboratory (SRL Inc, Tokyo, Japan). Serum CRP concentrations were measured by highly sensitive nephelometry at SRL Inc. In-house quality-control procedures were fulfilled at SRL Inc, and showed within- and between-assay coefficients of variation of 3.1% and 2.7%, respectively. The assay is sufficiently sensitive to detect 0.050 mg/L. Undetectable CRP values were recorded as 0.025 mg/L (n = 81). Serum CRP concentrations ≥1 mg/L were considered elevated for several reasons. First, previous studies have consistently shown that the median serum CRP concentration in Japanese (0.09-0.58 mg/mL) was lower than those in Western populations (1.5-6.2 mg/mL) [50,51]. Therefore to use cutoff value mainly based on Western population [17] might not be appropriate. Second, a higher serum CRP concentration is unfavorable for Japanese, although this value was not as high as those of other countries [50]. Third, because CRP concentration tends to increase with age [16], maintaining CRP concentrations as low as possible in younger age is an important preventive measure. Fourth, some previous Japanese studies used 1 mg/L of serum CRP concentration as cut off value [5,51].

### Other variables

Body height was measured to the nearest 0.1 cm with the subject standing without shoes. Body weight in light indoor clothes was measured to the nearest 0.1 kg. Body mass index (BMI) was calculated as body weight (kilograms) divided by the square of body height (meters). In the lifestyle questionnaire, the subject reported her residential area, which was grouped into one of three regions (north, Kanto and Tohoku; central, Tokai and Hokuriku; or south, Kyushu and Chugoku) and also into three categories according to population size (city with population ≥1 million, city with population <1 million, or town and village). Current smoking status (yes or no) was self-reported in the lifestyle questionnaire. Alcohol drinking (yes or no) and dietary supplement use (yes or no) were assessed as part of the DHQ. Physical activity was computed as average metabolic equivalent-hours per day [52] on the basis of the frequency and duration of five different activities over the preceding month, as reported in the lifestyle questionnaire.

### Statistical analysis

All statistical analyses were performed with SAS statistical software, version 9.2 (SAS Institute Inc., Cary, NC, USA). All reported *P* values are two-tailed, with a *P* value of <0.05 considered statistically significant. Dietary TAC intakes were adjusted for energy by the residual method using a regression model [53]. The percentage contribution of each individual food item to dietary TAC was calculated by dividing the TAC from each individual food item by the daily individual dietary TAC.

Significant differences between participants with normal and elevated CRP concentrations (cutoff 1.0 mg/mL) were assessed using Mann–Whitney signed-rank test or chi-square test. We used the non-parametric test because the result of Shapiro-Wilk’s W test for normality showed that all continuous variables had non-normal distribution. Associations with elevated serum CRP concentration were examined for energy-adjusted dietary TAC. Participants were divided into two categories according to the median value of dietary TAC. Odds ratios (ORs) and 95% confidence intervals (CIs) for elevated serum CRP concentrations were calculated after multivariate adjustment for potential confounding factors, including residential region, size of residential area, current smoking, alcohol drinking, dietary supplement use, physical activity (continuous), and BMI (continuous). Additionally, we adjusted for n-3 PUFA intake (energy-adjusted by residual method, continuous) because n-3 PUFA was the only dietary variable significantly associated with CRP; the following dietary variables were not associated with CRP: total fat, saturated fatty acids, monounsaturated fatty acids, polyunsaturated fatty acids, eicosapentaenoic acid + docosahexaenoic acid, alpha-linolenic acid, total dietary fiber, magnesium, vitamin C, fruit, vegetables, fish and shellfish, and dietary glycemic load [5]. Spearman’s correlation coefficients of each TAC and n-3 PUFA were 0.17 for FRAP, 0.17 for ORAC, 0.14 for TEAC, and 0.12 for TRAP. In terms of *trans* fatty acid, one previous study has shown the positive association with CRP [54]. We, however, did not investigate the association with CRP in the previous study. Nevertheless, while the major food contributors to *trans* fatty acid are fat and oil, bakery, confections, milk and milk products, and meat and meat products in young Japanese women (86%) [55], these foods seldom contributed to TAC as shown below (1-4%). Thus, it is unlikely that *trans* fatty acid and TAC are strongly correlated, neither does trans fatty acid confound or modify the association between TAC and CRP. We thus did not consider *trans* fatty acid in the present study.

## Results

Subject characteristics by normal and elevated CRP concentration are shown in Table 2. Median serum CRP concentration was 0.113 mg/L, with a range of 0.025 to 7.100 mg/L. The prevalence of elevated CRP concentration was 5.6%. Median dietary TAC was 10.61 mmol Fe^2+^/d for FRAP, 16.78 mmol TE/d for ORAC, 6.26 mmol TE/d for TEAC, and 6.71 mmol TE/d for TRAP. The range of Spearman’s correlation coefficients between each dietary TAC was 0.89-0.98 (data not shown). For the contribution of each food item to dietary TAC (Table 1), green, barley, and oolong tea was the major contributor (FRAP, 57%; ORAC, 47%; TEAC, 37%; and TRAP, 46%) and coffee was the second major contributor (FRAP, 3%; ORAC, 3%; TEAC, 6%; and TRAP, 10%) in all four assays. At food group level, non-alcoholic beverages, including green, barley, and oolong tea and coffee, was the major contributor in all food groups. Although vegetables was the second contributor in the food groups (FRAP, 8%; ORAC, 11%; TEAC, 9%; TRAP, 8%), it contributed less than single food item of green, barley, and oolong tea. The only significant difference between the elevated and normal CRP concentration groups was seen for dietary TAC (Table 2), with respective ratios for each dietary TAC of only 65-82% of those of the normal CRP concentration group.

**Table 2:** Characteristics of 443 Japanese women aged 18–22 years categorized by normal or elevated (cutoff 1 mg/L) serum CRP concentration*

	**Total (n = 443)**	**Normal CRP (CRP <1 mg/L) (n = 418)**	**Elevated CRP (CRP ≥1 mg/L) (n = 25)**	***P*†**
Serum CRP concentration (mg/L)	0.11 (0.06-0.23)	0.11 (0.06-0.20)	1.78 (1.29-3.06)	
Age (years)	19 (19–20)	19 (19–20)	19 (19–20)	0.72
Body mass index (kg/m^2^)	21.0 (19.5-22.5)	21.0 (19.5-22.6)	21.7 (19.3-22.2)	0.99
Residential region				0.37
North (Kanto and Tohoku)	268 (60.5)	250 (59.8)	18 (72.0)	
Central (Tokai and Hokuriku)	73 (16.5)	69 (16.5)	4 (16.0)	
South (Kyushu and Chugoku)	102 (23.0)	99 (23.7)	3 (12.0)	
Size of residential area				0.75
City with population ≥1 million	77 (17.4)	74 (17.7)	3 (12.0)	
City with population <1 million	329 (74.3)	309 (73.9)	20 (80.0)	
Town and village	37 (8.4)	35 (8.4)	2 (8.0)	
Current smoking				0.86
No	428 (96.6)	404 (96.7)	24 (96.0)	
Yes	15 (3.5)	14 (3.4)	1 (4.0)	
Alcohol drinking				0.10
No	248 (56.0)	230 (55.0)	18 (72.0)	
Yes	195 (44.0)	188 (45.0)	7 (28.0)	
Dietary supplement use				0.33
No	357 (80.6)	335 (80.1)	22 (88.0)	
Yes	86 (19.4)	83 (19.9)	3 (12.0)	
Physical activity (total metabolic equivalents-hours/d)	33.2 (32.2-34.5)	33.2 (32.2-34.4)	33.3 (32.4-34.6)	0.57
Energy intake (kcal/d)	1718 (1475–1986)	1717 (1476–1976)	1795 (1347–1994)	0.99
Total n-3 PUFA intake (g/d)‡	2.2 (1.9-2.6)	2.2 (1.9-2.6)	2.2 (1.9-2.6)	0.50
Dietary TAC‡				
FRAP (mmol Fe^2+^/d)	10.61 (7.42-16.06)	10.79 (7.55-16.33)	7.65 (5.47-11.68)	0.004
ORAC (mmol TE/d)	16.78 (12.43-24.12)	17.30 (12.93-24.63)	13.00 (9.38-16.93)	0.003
TEAC (mmol TE/d)	6.26 (4.67-8.83)	6.45 (4.73-8.92)	5.32 (3.66-6.01)	0.01
TRAP (mmol TE/d)	6.71 (4.34-10.24)	6.85 (4.50-10.48)	4.47 (3.60-6.48)	0.01

*CRP* C-reactive protein, *PUFA* polyunsaturated fatty acid, *TAC* total antioxidant capacity, *FRAP* ferric reducing ability of plasma, *ORAC* oxygen radical absorbance capacity, *TEAC* Trolox equivalent antioxidant capacity, *TRAP* total radical-trapping antioxidant parameter, *TE* Trolox equivalent.

* Values are medians (interquartile ranges) or numbers of subjects (%).

†*P* value between normal CRP and elevated CRP groups. For continuous values, the Mann–Whitney signed-rank test was used; for categorical values, the chi-square test was used.

‡ Energy adjustment was performed according to the residual method.

Table 3 shows the association between dietary TAC and elevated serum CRP concentration. The percentages of participants with elevated CRP levels in the low dietary TAC group (7.7-8.6%) were about two to three times higher than those in the high dietary TAC group (2.7-3.6%). ORs for elevated serum CRP concentration in the high versus low dietary TAC group were 0.30-0.45 in the crude model, and were hardly changed after adjustment for possible confounders. TAC from FRAP was inversely associated with serum CRP concentration (adjusted OR for elevated CRP concentration in high versus low dietary TAC group: 0.39 [95% CI: 0.16-0.98]; *P* = 0.04). With regard to TAC from ORAC, the inverse association between dietary TAC and serum CRP concentration was also observed with this method (adjusted OR: 0.48 [95% CI: 0.20-1.14]; *P* = 0.10), although the association did not reach statistical significance. TAC from TEAC was inversely associated with serum CRP concentration (adjusted OR: 0.32 [95% CI: 0.12-0.82]; *P* = 0.02). Additionally, TAC from TRAP was also inversely associated with serum CRP concentration (adjusted OR: 0.31 [95% CI: 0.12-0.81]; *P* = 0.02).

**Table 3:** Odds ratio and 95% confidence intervals for elevated (cutoff 1 mg/L) serum CRP concentrations by low or high dietary TAC groups in 443 Japanese women aged 18–22 years

	**Category of dietary TAC**	
	**Low (n = 221)**	**High (n = 222)**
FRAP (mmol Fe^2+^/d)*†	7.41 (0.71-10.60)	16.03 (10.61-37.17)
n (%) with elevated CRP	18 (8.1)	7 (3.2)
Crude model	1 (ref)	0.37 (0.15-0.90)
Multivariate model‡	1 (ref)	0.39 (0.16-0.98)
ORAC (mmol TE/d)*†	12.43 (1.18-16.73)	24.13 (16.78-50.77)
n (%) with elevated CRP	17 (7.7)	8 (3.6)
Crude model	1 (ref)	0.45 (0.19-1.06)
Multivariate model‡	1 (ref)	0.48 (0.20-1.14)
TEAC (mmol TE/d)*†	4.67 (0.44-6.25)	8.83 (6.26-31.75)
n (%) with elevated CRP	19 (8.6)	6 (2.7)
Crude model	1 (ref)	0.30 (0.12-0.75)
Multivariate model‡	1 (ref)	0.32 (0.12-0.82)
TRAP (mmol TE/d)*†	4.34 (0.31-6.66)	10.23 (6.71-52.42)
n (%) with elevated CRP	19 (8.6)	6 (2.7)
Crude model	1 (ref)	0.30 (0.12-0.75)
Multivariate model‡	1 (ref)	0.31 (0.12-0.81)

*CRP* C-reactive protein, *TAC* total antioxidant capacity, *FRAP* ferric reducing ability of plasma, *ORAC* oxygen radical absorbance capacity, *TEAC* Trolox equivalent antioxidant capacity, *TRAP* total radical-trapping antioxidant parameter, *TE* Trolox equivalent; ref, reference.

* Values are medians (ranges).

† Energy adjustment was performed according to the residual method.

‡ Adjusted for residential region [north (Kanto and Tohoku), central (Tokai and Hokuriku), and south (Kyushu and Chugoku)], size of residential area (city with a population ≥1 million, city with a population <1 million, and town and village), current smoking (yes or no), alcohol drinking (yes or no), dietary supplement use (yes or no), physical activity level (total metabolic equivalents-hours/d, continuous), body mass index (kg/m^2^, continuous), and n-3 polyunsaturated fatty acid intake (g/d, energy-adjusted, continuous).

## Discussion

In this study, we found that a higher dietary TAC was associated with a lower prevalence of elevated CRP concentration among young Japanese women. Dietary TAC from FRAP, TEAC, and TRAP were significantly inversely associated with serum CRP concentration. Dietary TAC from ORAC also showed an inverse association with serum CRP. The dietary TAC values of elevated serum CRP concentration group were significantly lower than those of normal CRP group. To our knowledge, this is the first study to examine the association between dietary TAC and elevated CRP concentration in a non-western population.

In the present study, dietary TAC was estimated using a DHQ for the four different assays of FRAP, ORAC, TEAC, and TRAP. The TRAP databases [15,33] contain the TAC values of only a limited number of foods, and those of 38 foods could not be obtained. In contrast, the FRAP databases [29-32] contain an extensive number of foods, and values could not be obtained for only 16 foods. Further, most foods in the FRAP assay were assigned an analytical rather than a substituted or calculated TAC value. These different results were probably due to the different antioxidant mechanisms derived from different substrates, reaction conditions, and quantification methods [56]. Nevertheless, the major sources of dietary TAC were the same among the TAC assays and the TAC values of these food items were available in the literature (i.e., teas, coffee, and chocolate). Our study showed that dietary TAC from FRAP, TEAC, and TRAP were significantly inversely associated with serum CRP concentration. Dietary TAC from ORAC also showed an inverse association with serum CRP, albeit without statistical significance. Additionally, the correlation coefficients between respective dietary TAC values were high. These results may suggest that dietary TAC was inversely associated with serum CRP concentration regardless of assay.

Whereas previous western studies showed that the main contributors of dietary TAC were coffee, fruits, vegetables, and alcohol beverages [57-60], the major contributor in this present young Japanese population was green, barley, and oolong tea. Even the sum contributions of all vegetables or fruits were less than the contribution of single green, barley, and oolong tea. Additionally, vegetables commonly consumed in Japan differ from those in Italy [14,15], and fruit and vegetable items consumed by contemporary young Japanese women differ from those by young Spanish adults [12]. Nevertheless, dietary TAC was inversely associated with CRP in our Japanese population, as with Italian and Spanish populations [10,12], suggesting that a high dietary TAC is important for a low prevalence of elevated CRP level regardless of the type or origin of food. The present finding of a significant association between dietary TAC and CRP contrasts with a previous study in the same population which found no association between single intakes of vitamin C, fruits, and vegetables and serum CRP [5]. This difference in turn suggests that complex combinations of antioxidant nutrients and foods might be more strongly associated with CRP than any single nutrient or food alone.

Dietary TAC was significantly associated with some lipid biomarkers (e.g., oxidized low-density lipoprotein; ox-LDL) and plasma TAC was negatively correlated with ox-LDL concentrations [61]. These results may suggest that high consumption of antioxidant-rich foods decrease oxidation in the low-density lipoprotein by increasing the plasma TAC availability. These favorable situations might relate to low serum CRP concentrations. CRP production in the liver is induced by interleukin-6 (IL-6) [1,62]. Adipocytes produce many inflammatory cytokines, including IL-6, and their transcription is regulated by the nuclear transcription factor-κB (NF-κB) [62,63]. Given previous findings that dietary TAC was inversely associated with CRP as well as with mRNA expression of NF-κB subunit-1 and IL-6 in young Spanish adults [12] and that ox-LDL is able to induce a pro-inflammatory status by the activation of NF-κB [61], the association between dietary TAC and CRP might accordingly relate to NF-κB-regulated pathways interacting ox-LDL. However, that study also showed that dietary TAC was not associated with serum IL-6 concentration [12]. Meanwhile, another study reported that dietary TAC tended to show an inverse association with IL-6 level [11]. These inconsistent results hamper any comprehensive understanding of the mechanism of the relationship between lower CRP concentrations and dietary TAC. Various antioxidants contribute to dietary TAC and these compounds regulate inflammation via multiple signaling pathways [64]. The mechanism by which dietary TAC associates with serum CRP concentration is therefore likely complex. Further, the validity of TAC as a measure of functional efficacy of antioxidant defense in vivo was questioned. In fact, not only dietary phytochemicals, but also powerful enzymes in cells and tissues contribute to the prevention of oxidation. Additionally, antioxidant capacity of some molecules in foods may change into the uptake and metabolism [65]. We should take into account the fact that dietary TAC may not necessarily reflect antioxidant level in vivo.

Several limitations of the present study should be mentioned. First, because TAC data on Japanese foods were available from only a single database [14], dietary TAC was estimated using databases developed in other countries. Additionally, many foods were assigned a substituted or calculated TAC value. Further, because a reliable TAC database for dietary supplements could not be obtained, we did not consider the intake of dietary supplements in calculating dietary TAC. Second, the DHQ was not specifically designed to measure dietary TAC. In assessing dietary TAC, we were unable to investigate the validity of the DHQ against the 16-day dietary records we previously used to investigate the validity of other dietary variables [20,21] because the dietary record contained an insufficient number of foods with information on TAC values (n = 143–373). However, a previous validation study among 92 adult women reported Pearson correlation coefficients of 0.64 for β-carotene, 0.52 for vitamin C, and 0.47 for α-tocopherol [21], and Spearman correlation coefficients for food groups were 0.75 for coffee, 0.59 for green and oolong tea, 0.56 for total vegetables, and 0.40 for fruits [20]. This satisfactory validity of the DHQ for a wide range of antioxidant nutrients and foods provides some reassurance. Third, participants of the present study were selected female dietetic students, not a random sample of Japanese women. In addition, because of our recruitment procedure, the exact response rate was unknown, which might have produced recruitment bias. Thus, our results cannot easily be extrapolated to the general Japanese population. Fourth, although we attempted to adjust for a wide range of potential confounding variables, we are unable to rule out residual confounding. Finally, the cross-sectional nature of the study hampers the drawing of any conclusions on causal inferences among dietary TAC and serum CRP concentration.

## Conclusion

This study showed that dietary TAC was inversely associated with serum CRP concentration among young Japanese women regardless of assay. This result will provide valuable insights from a preventive perspective. Our result should be confirmed in other Asian and Japanese populations.

## Abbreviations

TAC: Total antioxidant capacity; CRP: C-reactive protein; DHQ: Diet history questionnaire; PUFA: Polyunsaturated fatty acid; FRAP: Ferric reducing ability of plasma; ORAC: Oxygen radical absorbance capacity; TEAC: Trolox equivalent antioxidant capacity; TRAP: Total radical-trapping antioxidant parameter; TE: Trolox equivalent; BMI: Body mass index; OR: Odds ratio; CI: Confidence interval; IL-6: Interleukin-6; NF-κB: Nuclear transcription factor-κB.

## Competing interests

The authors declare that they have no competing interests.

## Authors’ contribution

SK formulated the hypothesis, analyzed and interpreted the data, and wrote the manuscript. KM contributed to the concept and design of the study, the study protocol, and data collection and management; coordinated the field work; interpreted the data; and contributed to the writing and editing of the manuscript. SS was responsible for the concept and design of the study, the study protocol, and data collection and management; and contributed to the writing and editing of the manuscript. KU contributed to the concept and design of the study, the study protocol, and data collection. MY, HH, TG, JO, KB, KO, TK, RW, and YS contributed to data collection. All authors contributed to the final version of the manuscript. All authors read and approved the final manuscript.
